# Bioactive Nanostructured Scaffold-Based Approach for Tendon and Ligament Tissue Engineering

**DOI:** 10.3390/nano13121847

**Published:** 2023-06-12

**Authors:** Darshan Tagadur Govindaraju, Chih-Hao Chen, K. T. Shalumon, Hao-Hsi Kao, Jyh-Ping Chen

**Affiliations:** 1 Department of Chemical and Materials Engineering, Chang Gung University, Kwei-San, Taoyuan City 33302, Taiwan; darshu.tg@gmail.com; 2 Department of Plastic and Reconstructive Surgery, Chang Gung Memorial Hospital at Keelung, Chang Gung University College of Medicine, Anle, Keelung 20401, Taiwan; chchen5027@gmail.com; 3 Craniofacial Research Center, Chang Gung Memorial Hospital at Linkou, Kwei-San, Taoyuan City 33305, Taiwan; 4 Department of Chemistry, Sacred Heart College, Mahatma Gandhi University, Kochi 682013, India; shaumon@gmail.com; 5 Division of Nephrology, Chang Gung Memorial Hospital at Keelung, Chang Gung University College of Medicine, Anle, Keelung 20401, Taiwan; kao95812@yahoo.com.tw; 6 Department of Neurosurgery, Chang Gung Memorial Hospital at Linkou, Kwei-San, Taoyuan City 33305, Taiwan; 7 Research Center for Food and Cosmetic Safety, College of Human Ecology, Chang Gung University of Science and Technology, Kwei-San, Taoyuan City 33305, Taiwan; 8 Department of Materials Engineering, Ming Chi University of Technology, Tai-Shan, New Taipei City 24301, Taiwan

**Keywords:** tissue engineering, scaffold, electrospinning, tendon, ligament, growth factors, dynamic culture

## Abstract

An effective therapeutic strategy to treat tendon or ligament injury continues to be a clinical challenge due to the limited natural healing capacity of these tissues. Furthermore, the repaired tendons or ligaments usually possess inferior mechanical properties and impaired functions. Tissue engineering can restore the physiological functions of tissues using biomaterials, cells, and suitable biochemical signals. It has produced encouraging clinical outcomes, forming tendon or ligament-like tissues with similar compositional, structural, and functional attributes to the native tissues. This paper starts by reviewing tendon/ligament structure and healing mechanisms, followed by describing the bioactive nanostructured scaffolds used in tendon and ligament tissue engineering, with emphasis on electrospun fibrous scaffolds. The natural and synthetic polymers for scaffold preparation, as well as the biological and physical cues offered by incorporating growth factors in the scaffolds or by dynamic cyclic stretching of the scaffolds, are also covered. It is expected to present a comprehensive clinical, biological, and biomaterial insight into advanced tissue engineering-based therapeutics for tendon and ligament repair.

## 1. Tissue Engineering of Tendon and Ligament

**Figure 1:**
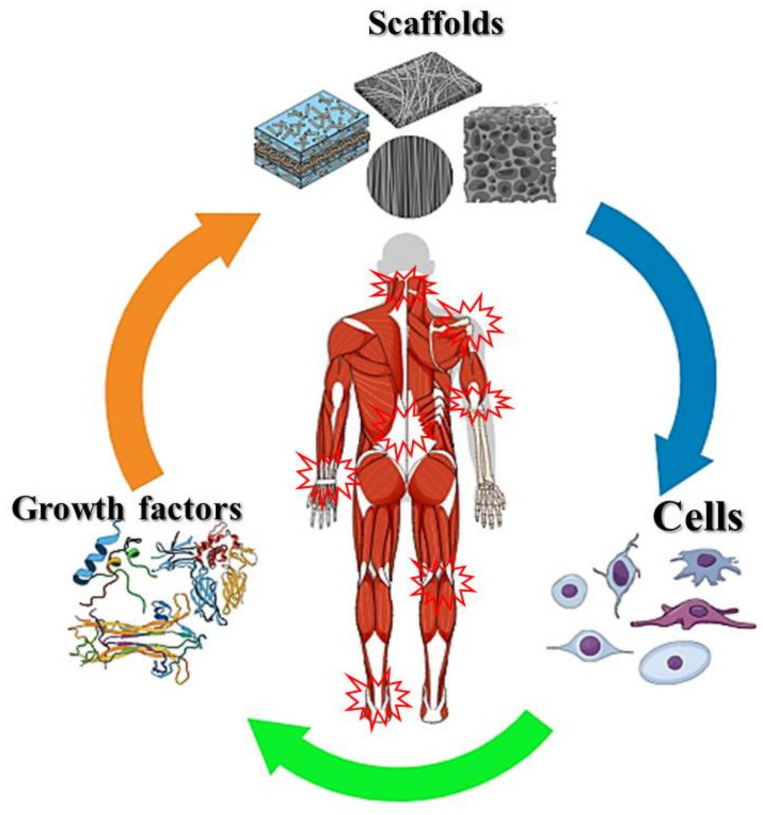
The three important components to be considered for the application of tissue engineering in damaged tissues repair. A coordination of cells cultured in a biomaterial-based scaffold, which is endowed with suitable biochemical signals provided by growth factors, can regenerate tissues in vitro and repair damaged tissues in vivo.

Tissue engineering is an evolving multidisciplinary field for regenerating damaged tissue/organs by transplanting a cell-seeded functional scaffold. Using converged knowledges from biology, materials science, chemistry, and engineering, tissue engineering has combined the use of cells, growth factors, and scaffolds to make remarkable progress over the last decade (Figure 1) [1]. Within the three components, the scaffold is the most important one; it interacts with cells and growth factors and provides a structural support for cell attachment and tissue development [2]. To facilitate cell attachment, growth, and differentiation, the scaffold should be endowed with unique properties, such as biocompatibility, biodegradability, and a degradation rate that can match the rate of tissue regeneration [3]. The extracellular matrix (ECM) is secreted by cells to provide a structural support for cells. It also contains many highly regulated biologically active molecules that can determine the action and fate of cells [4]. The proteins and glycosaminoglycans (GAGs) are types of biomolecules found in the ECM, and they include fibrous proteins, adhesive glycoproteins, and proteoglycans. The proteoglycans consist of a core protein and covalently attached GAG chains [5]. The fibrous proteins can provide tensile strength while the proteoglycans can resist compressive forces [6]. Although the composition of ECM may be different, cell adhesion, cell-to-cell communication, and cell differentiation are the common functions of the ECM. The ECM can bind, release, and activate signaling molecules, and also modify cellular response to soluble factors [7]. Therefore, the scaffold should mimic the morphological structure and chemical composition of the ECM, allowing cells to attach to the scaffold, proliferate, and differentiate into new tissues [8].

### 1.1. Structure of Tendons and Ligaments

**Figure 2:**
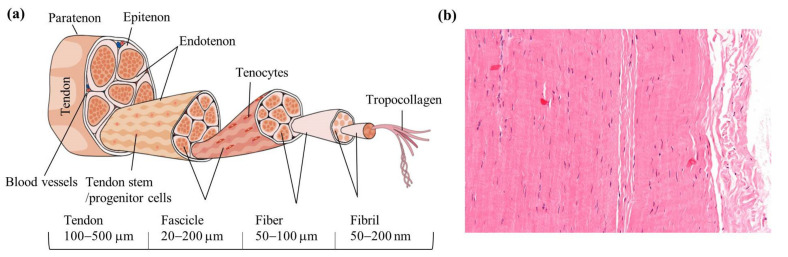
The anatomical structure (**a**) and the histological image (**b**) of a tendon.

The tendons and ligaments are fibrous, dense, regular connective tissue, allowing for movement and maintenance of body posture. The main functions of tendons and ligaments are to connect and transmit forces from muscles to bones and from one bone to another, respectively. They share a comparable composition and hierarchical structure. The ECM of tendons is composed predominantly of collagen, which accounts for up to 85% of the dry weight of a tendon tissue. Within different types of collagen in tendon ECM, collagen type I represents ~95% of the dry weight, with the rest being collagen type III, V, XI, XII, and XIV. The tendon is composed of two primary cellular components: tenocytes that are fully differentiated cells and account for 90–95% of cellular content in a healthy mature tendons, and tendon stem/progenitor cells that are located within the ECM of the tendon. Additional cellular components include synovial cells located on the surface and vascular cells present in the endotenon and epitenon regions. In normal circumstances, the maintenance and repair of tendons are carried out by tenocytes, but tendon stem/progenitor cells also perform a crucial function in this process by engaging themselves in self-renewal and differentiation into tenocytes [9]. Structurally, a tendon has a well-organized structure with three layers of fibrous bundles: the primary fiber bundle (sub-fascicle) formed from a bunch of collagen fibers, the secondary fiber bundle (fascicle) formed from a group of primary bundles, and the tertiary bundles formed from a bundle of secondary fiber bundles (Figure 2) [10,11]. The diameters of the sub-fascicles and fascicles are dependent on the overall length of the tendon, but small tendons (such as flexor and extensor tendons in the fingers and toes) have smaller sub-fascicles and fascicles, while large tendons (such as the Achilles tendons) have larger sub-fascicles and fascicles. In humans, the fascicles have diameters ranging from 150 to 1000 µm, while the tertiary fiber bundles have diameters ranging from 1 to 3 mm [12].

**Figure 3:**
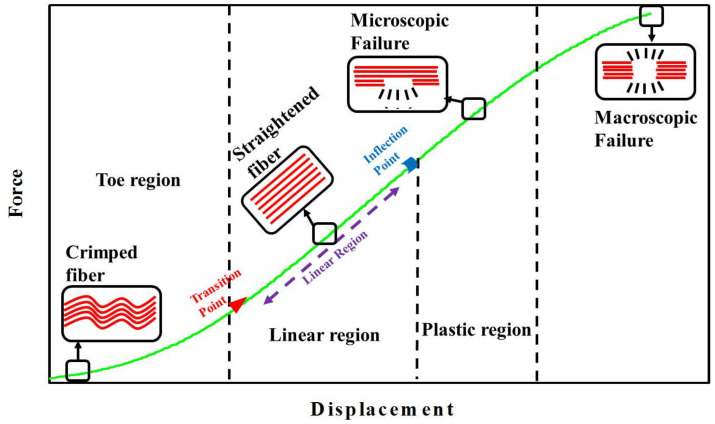
A typical force vs. displacement curve of a tendon and its collagen fiber structure during stretching.

The structure and composition of a tendon leads to its unique ability to withstand a high tensile strength, and this distinct mechanical characteristic distinguishes it from other tissues in the body. Indeed, in order to play a key function in joint stability during movement and physical activity, the tendon and ligament need to display superior mechanical strength and flexibility [13,14,15]. This can be revealed from the four distinct regions in a typical force vs. displacement curve of a tendon (Figure 3) [16]. However, it should be noted that although the typical force-displacement curves of tendons and ligaments show similar profiles, these tissues show different displacement values at the end of the toe region as well as with different failure forces. The collagen fibers in a tendon or ligament are crimped while at rest and start to align with each other when a load is applied, and this leads to the loss of the crimped structure found in the toe region. The second region is referred to as the elastic/linear region as all recruited collagen fibers are straightened when a tendon is stretched in this region. In this region, the tissue provides excellent elastic recovery when the load is removed, and the fibers gradually begin to slide in relation to one another. The slope of the curve changes when the force is raised beyond the linear region, at the end of which the plastic region begins. Continued straining causes microscopic tears in the tissue, which can result in tendinopathy. As the fibers are subjected to further stress/load, the macroscopic failure finally appears and eventually tendon rupture occurs [17]. An inflection point in the stress–strain curve represents the start of the damage to the tendon, while an inflection point in the stress–strain curve marks the point from strain-stiffening to strain-softening, and may indicate a damage threshold [18]. The mechanical properties of tendons and ligaments are closely associated with their cross-sectional areas and physiological roles, as well as the rate of deformation during loading.

The musculoskeletal tissues contain both soft and hard tissues, which provide a gradient of mechanical properties with progressive transition from one end to the other. To meet this need, material gradients existed at the junctions for reducing stress concentrations found at these sites. At the interface of tendon/ligament to muscle or bone, the junctions are called entheses and myotendinous junctions, which enable a physiological transmission of load over the junction over a wide area [19,20]. The entheses connect tendons/ligaments to bones and vary widely based on the anatomical sites and the structures involved, but in general they can be divided into two main categories: the fibrous entheses and the fibrocartilaginous entheses. In a fibrous enthesis, tendons/ligaments are connected to bones through acute angles with Sharpey’s fibers and collagen fibers extending directly from the periosteum [21]. In contrast, the fibrocartilaginous enthesis contains a progressive mineralization gradient, which can be separated into four zones: tendon/ligament, unmineralized fibrocartilage, mineralized fibrocartilage, and bone. The complex and heterogeneous structure of the enthesis is essential to ensure smooth mechanical stress transfer between bones and tendons/ligaments. As the interface is not regenerated following injury and usually results in high rupture recurrence rates, the tissue engineering approach represents a promising strategy for the regeneration of a functional enthesis [22,23]. The interface between the tendon and the muscle tissue is a myotendinous junction, which offers a gradual transition between stiff tendons to soft muscles. Different to entheses, a myotendinous junction connects a high cellularity tissue (muscle) to a low cellularity ECM-rich tissue (tendon). The microscopic structure of a myotendinous junction shows myofibers that can generate conical finger-like projections interlocking the tendon ECM [24]. At the macroscale, it creates a network overlapping the muscle and tendon tissues and increases their interface area. Sensini et al. have provided a comprehensive review of notable works using electrospun scaffolds for tendon and ligament tissue engineering at the enthesis and myotendinous junction [25].

### 1.2. Healing of Injured Tendon

As tendons and ligaments have a unique structure and function, their repair after injury remains a challenging task for clinicians. The low cellularity and hypovascularity in these tissues usually leads to ineffective treatment and reduced healing ability [26]. Indeed, tendon and ligament injuries are among the most prevalent types of injuries sustained by the body, especially in the young and physically active population. Apart from common injuries, the elderly are often affected by chronic deterioration of these tissues, which occurs as a result of aging. Annually, around 300,000 surgical repairs of tendons in the hand, foot, and ankle, and roughly 350,000 anterior cruciate ligament (ACL) reconstructive procedures are performed in the United States, costing approximately USD 30 billion dollars [27]. Tendon injuries may occur from acute or chronic changes, or a combination of both. Chronic injuries are often related to intrinsic factors such as genetics, sex, age, nutrition, and general health, while acute injuries are commonly associated with extrinsic factors such as excessive and inadequate mechanical loadings [28,29]. As mentioned before, the low cellularity, hypovascularity, and low metabolic activity of a tendon make its ability to heal after injury particularly poor, and in most cases the repaired tendon cannot fully restore its mechanical properties as before, and re-rupture is likely to occur. This problem could be linked to insufficient tissue regeneration, where substantial differences in molecular and histological structure between neo-tendon and native tendon are commonly found [30]. This situation may arise from several reasons, such as change in tenocyte phenotype as well as change in ECM composition and arrangement in the neo-tissue [31]. All these problems eventually lead to weakness of regenerated tissue, with post-operative discomfort and fibrous adhesion, and eventually results in tearing or complete rupturing of tendons [32].

**Figure 4:**
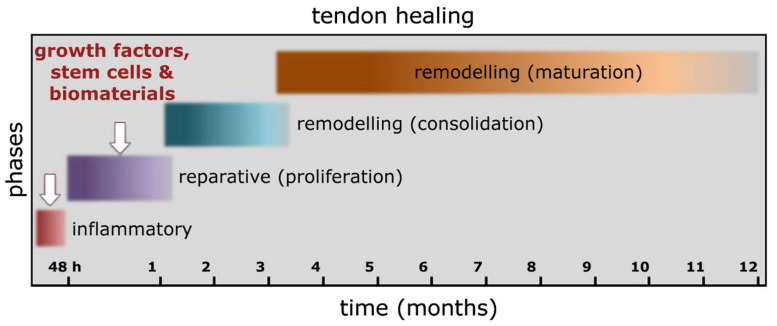
Overview of the tendon repair process in humans. The healing of ruptured tendons passes through three phases with distinctive cellular and molecular cascades. Their duration depends on the location and severity of the injury. During the first two stages (indicated by white arrows), growth factors, stem cells or biomaterials are implemented. Reproduced with permission from [35]. Copyright 2015. Elsevier.

There are three main stages during tendon healing post-injury, including inflammation, proliferation, and remodeling (Figure 4) [33]. After tendon injury, the next 3 to 7 days are the inflammation phase. During this period, various chemotactic agents are released, modulating the inflammatory responses, stimulating the proliferation of fibroblasts and tenocytes, activating the angiogenesis process, and synthesizing mostly type III collagen. The proliferation phase, which is the second phase, usually lasts until the third or fourth week post-injury and is characterized by a rapid increase in cellularity by fibroblasts, as well as by random ECM structures. The last remodeling phase can be further subdivided into two stages: consolidation and maturation. During the consolidation stage, the production of collagen and GAGs is reduced. At the same time, the tenocytes and collagen fibers begin to align themselves along the longitudinal axis of the tendon, and finally become capable of bearing load. At this time, the cellularity of the tissue decreases, while production rate of type I collagen increases. The maturation stage is a lengthy process that may take up to a year to complete. During this period of tendon healing, the scar tissue gradually starts to show and a histological appearance that is more resemblance of a healthy tendon may appear as the healing process progresses and tendon tissue matures. However, the biomechanical properties of repaired tendons are usually weaker than those of the native tendons, and collagens in the repaired tendons usually have less crosslinking and have smaller diameters than those in undamaged tendon, rendering the tendon more vulnerable to re-injury [34].

### 1.3. Treatment of Tendon and Ligament Injuries

Tendon and ligament injuries can be treated with extracorporeal shockwave therapy, eccentric exercise therapy, ultrasound, and low-intensity laser therapy, all of which are considered conservative and unsatisfactory [36]. Additionally, the use of a non-steroidal anti-inflammatory drugs for pain management is controversial because there is a high risk of spontaneous tendon and ligament rupture after taking these medicines [37]. Tendon pathology can be mainly divided into tendon laceration or tendinopathy [38]. Tendon lacerations are commonly treated with advanced suturing techniques; however, initial problems such as repair site rupture and postoperative adhesions may persist despite improved surgical methods and rehabilitation approaches [39]. In cases where substantial tissue defects occur, tendon and ligament grafting may be necessary by employing autografts or allografts [40,41]. Allografts have several limitations, including the high risk of infectious disease transmission, potential immune response from the recipient, and the difficulty of preservation during transportation [42]. Autografts are used widely in clinical practice for tendon repair with excellent long-term results when compared to allografts, but problems such as donor site morbidity and lack of autograft material still persist [43]. Furthermore, when graft efficiency is low during the tendon/ligament restoration process, the graft cannot meet the mechanical strength requirements [44]. A significant amount of emphasis is therefore being paid to the fabrication of scaffolds made from degradable natural or synthetic polymers for the treatment of tendon/ligament injuries by tissue engineering. After implantation of the cell/scaffold construct, the tissue engineering approach for tendon/ligament repair could be accomplished pending successful regeneration of functional neo-tendon/ligament tissues to solve the problems faced by tissue grafting, which usually fail to restore the lost functionality.

## 2. Preparation of Scaffolds for Tendon and Ligament Tissue Engineering

### 2.1. Scaffold Materials

Various polymers have been employed as scaffold materials for tendon and ligament tissue engineering [45,46,47,48,49,50,51,52]. Scaffolds produced from biodegradable natural polymers or synthetic polymers such as gelatin [45], collagen [53], chitosan, cellulose [51], poly(lactic acid) (PLA), poly(lactic-co-glycolic acid) (PLGA), poly(glycolic acid) (PGA) [54], and poly(caprolactone) (PCL) [46] have been studied before with promising results. Synthetic and natural polymers have their own abilities and inabilities. Synthetic polymers have controllable properties and good mechanical strength but poor biocompatibility; on the other hand, natural polymers can promote cell proliferation and attachment and can also mimic the native ECM components. The main drawbacks of natural polymers are their low mechanical strength and fast degradation rates [55]. Overcoming these drawbacks is possible by combining two or more polymers to create a scaffold with desirable properties [47,48,52,54,56]. However, it should be noted that we do not differentiate biomaterials that can be used as augmentation strategies or as tendon substitutes for tendon/ligament repair in the following review.

#### 2.1.1. Synthetic Polymers

Synthetic polymers have been widely used to produce tendon/ligament scaffolds due to their availability, ease of processing, and reproducibility [10,14]. In addition, synthetic polymers exhibit low immunogenicity and are more versatile with controllable chemical and physical properties to meet different requirements by the scaffolds [57,58]. Because of their mechanical strength and biodegradability, synthetic polymers such as PLA, PCL, PGA, PLGA, and poly(L-lactide-co-caprolactone) (PLCL) are widely used in tissue engineering applications [57,59]. Moreover, these polymers have been approved by the US Food and Drug Administration (FDA) for certain human uses. However, they still lack proper biological cues for cell adhesion and proliferation, which must be overcome by blending or coating with natural polymers [60,61].

Poly(caprolactone) (PCL) is a linear hydrophobic polymer with good mechanical properties, a low melting point, and good processability [62,63]. It is used for a variety of biomedical applications in humans, including as a suitable candidate to fabricate fibrous scaffolds [56,64]. However, the PCL is hydrophobic in nature, which may reduce its ability to induce cell adhesion, proliferation, and differentiation. Pouly et al. produced random and aligned PCL nanofibers in flat sheets and rolled them into nanofiber bundles that could mimic the size scale of fascicle units in soft musculoskeletal tissues and bear tensile loads [65]. The geometry and orientation of aligned nanofiber can significantly increase the strength of the scaffold, with aligned fiber sheets and bundles showing modulus 125% and 45% higher than random nanofiber sheets and bundles, respectively. In addition, the yield stress and yield strain of aligned nanofiber bundles were 107% and 140% higher than those of aligned nanofiber sheets. The adipose derived stem cells (ADSCs) were cultured in these nanofiber scaffolds over a period of 7 days, with cells attaching preferentially along the fiber axis in aligned nanofiber bundles. Overall, the PCL scaffold has potential for ligament and tendon tissue engineering and can provide a milieu for cell adhesion, proliferation, and elongation, as well as providing mechanical strength from fiber bundles [65]. Bosworth et al. fabricated yarn scaffolds based on PCL fibers and seed human mesenchymal stem cells (MSCs) in the scaffolds for cell culture in both dynamic and static modes [66]. The yarn fiber porosity was ~56%, and the scaffold provided a suitable macroporous structure to support cell infiltration. The dynamically loaded PCL fiber yarn scaffold showed a thicker cell layer around the scaffold’s exterior than the statistically cultured yarn, with cell proliferation and ECM deposition occurring predominantly along the axial cell orientation direction after 21-day in vitro culture. The dynamic cultured fibrous yarn had the highest tensile strength, as well as up-regulated specific gene marker expression; endorsing the electrospun yarn scaffold can promote seeded MSCs to differentiate towards a tendon lineage [66]. In a different study, aligned and nonaligned multilayered PCL fibrous scaffolds were seeded with human ADSCs. The scaffolds showed cell infiltration and collagen deposition throughout the thickness of the scaffold, as depicted from scanning electron microscopy (SEM) images after 28-day in vitro culture [67]. Most importantly, the aligned multilayered PCL scaffold was found to enhance collagen alignment and tendon-related gene expression and construct mechanical properties when compared with nonaligned multilayered scaffolds. To introduce tendon-specific biochemical cues into electrospun PCL scaffolds, three PCL single yarns composed of aligned PCL nanofibers were braided to form a multi-yarn scaffold. The electrospun multi-yarn fibrous scaffold was surface modified with oxygen plasma, conjugated with heparin and immobilized with fibrous growth factor-2 (FGF2) for the culture of tendon-derived fibroblasts. The biochemical cues from FGF2 as well as the physical cues from aligned fibrous structure can accelerate cell proliferation, increase ECM synthesis, and promote tendon maturation [68]. Dynamic culture in a bioreactor under uniaxial cyclic tensile loading with 5% strain further increased the rates of cell proliferation and tenogenic differentiation, and the cell/scaffold construct demonstrated superior mechanical properties and tendon regeneration capabilities 6-week post-implantation to repair extensor digitorum tendon defects. However, it should be noted that a scaffold fabricated from PCL may have limited tensile strength and a slow degradation rate when compared to other polymers used in tendon and ligament tissue engineering [59,69].

Poly(lactic acid) (PLA) is a linear aliphatic polymer, with lactide being the monomer component [70,71]. It undergoes hydrolytic degradation in aqueous solutions by randomly cleaved ester bonds into lactic acid. The degradation rate is controlled by the amount of absorbed water, the solubility of degradation products, and the diffusion coefficients of chain fragments within the polymer [72]. The lactic acid could be eliminated from the body by incorporation into the tricarboxylic acid cycle, which is mainly eliminated through respiration by the lungs as CO_2_. However, it undergoes slow degradation within a period of 10 months to 4 years [73]. PLA has been mainly used as a scaffold material for tendon/ligament engineering based on its molecular weight, crystallinity, shape, and site of impact [73,74,75,76,77,78]. Sensini et al. cultured human fibroblasts cells on structured bundles of fibrous PLA/collagen scaffolds and found the electrospun scaffolds showed weaker mechanical properties, such as stiffness, strength, and toughness, after ageing in phosphate buffer saline (PBS) [79]. However, increasing the mass ratio of PLA in the scaffold improves mechanical properties such as stiffness and ductility. Cooper et al. fabricated fibrous scaffolds by braiding PLA fibers using a 3D braiding machine and seeded the scaffolds with four different types of connective tissue cells from anterior cruciate ligament (ACL), medical collateral ligament (MCL), Achilles tendon (AT), and patellar tendon (PT) [74]. Their study revealed that ACL cells showed the highest cell proliferation rates and gene expression of fibronectin, collagen type I (COL I), and collagen type III (COL III), followed by PT and AT cells during in vitro culture. Their study suggested that the ACL is the most suitable cell source for tissue-engineered ligament [74]. Jenkins et al. proposed biodegradable scaffolds from aligned melt-blown PLA fabrics as scaffolds for rotator cuff tendon tissue engineering [80]. The PLA scaffold demonstrated mechanical anisotropy for the attachment, proliferation, and spreading of human ADSCs. The ADSC-seeded scaffolds showed a significant decrease in Young’s modulus and loss of integrity as found in cell-free scaffolds over 28 days. However, the DNA, sulfated glycosaminoglycans (GAGs), and collagen content significantly increased during this period, and histology and polarized light microscopy demonstrated deposition of aligned collagen fibers in the scaffolds [80]. Considering PLA is a hard and brittle polymer, which may not be suited for soft tissue applications as in tendons, Vuornos et al. braided filaments fabricated from a PLA/PCL copolymer PLCL into fibrous scaffolds for the efficient production of a tendon-like matrix in vitro using human ADSCs [81]. However, they reported that the braided PLA scaffold had higher cell adhesion, proliferation, and tenogenic differentiation ability compared to a PLCL scaffold by significantly enhancing tendon-like matrix production in a tenogenic medium. Furthermore, total collagen content and tendon-specific gene markers were also significantly higher using a PLA scaffold, which provided an elastic modulus as high as that of a native Achilles tendon [81].

Poly(lactic-co-glycolic acid) (PLGA) is a linear aliphatic polymer formed from both lactide and glycolide monomers and has been approved by the FDA for a wide range of clinical applications [70,82]. For tendon and ligament tissue engineering, the PLGA scaffold has attracted particular attention for its design flexibility, good mechanical strength, and complete bioresorption ability in vivo [70,83,84,85,86]. Sahoo et al. fabricated a nano-micro fibrous PLGA scaffold by deposition of electrospun PLGA nanofibers onto the surfaces of a knitted PLGA scaffold for tendon/ligament tissue engineering [84]. The bone marrow stromal cells from pigs were seeded by directly pipetting a cell suspension or by immobilizing in a fibrin gel matrix coated to the knitted PLGA scaffold. Although the cell attachment was comparable, faster cell proliferation and up-regulation of specific gene markers such as COL I, decorin, and biglycan was found by direct pipetting. Although many studies on 3D braided scaffolds have been carried out for anterior cruciate ligament (ACL) replacement, an optimized material selection based on cellular response remains a challenging issue to be solved [87,88]. Towards this, Lu et al. used in vitro study to optimize a braided fibrous scaffold for ACL regeneration by focusing on the influence of composition of three synthetic poly-α-hydroxyester fibers, poly(glycolic acid) (PGA), poly(L-lactic acid) (PLLA), and poly(lactic-co-glycolic acid) (PLGA), on scaffold mechanical properties, degradation, and cellular response [85]. The scaffolds were pre-coated with fibronectin, an important protein that is upregulated during ligament healing, prior to in vitro culture with rabbit ACL cells. Within the three scaffolds, the PGA scaffold showed the highest tensile strength, and the PLGA scaffold showed the lowest, but the PGA scaffold showed rapid degradation and resulted in matrix disruption and cell death over time. Overall, the fibronectin-coated PLLA scaffold was found to be the most suitable substrate for ACL tissue engineering based on overall cellular response, mechanical properties, and degradation in vitro [85]. Ouyang et al. examined a knitted PLGA scaffold for applications in tendon regeneration in a rabbit model with 10-mm Achilles tendon gap defects. They observed regenerated tendon tissues containing COL I and Col III as early as 2 weeks post-implantation [89]. Additionally, at 12 weeks post-implantation, both the tensile stiffness and the modulus of the regenerated tendon reached ~50% that of a normal tendon, with even better results achieved for scaffolds seeded with bone marrow stromal cells. However, as knitted scaffolds require a coated gel layer from fibrin or collagen for cell seeding, it may not be used for ligament reconstruction in the knee joint, because the cell-loaded gel will be dissociated from the scaffold during motion.

#### 2.1.2. Natural Polymers

Natural polymers are gaining attention in biomedical applications due to their unique properties, such as biocompatibility, and as components in the ECM [71]. These polymers are recognizable in a biological environment because they have a similar composition to macromolecules found in vivo, which provides them a physiologically related degradation rate [90,91]. In addition, natural polymers contain many functional groups for conjugation with growth factors, which is beneficial for application in tendon/ligament tissue engineering. Even with these benefits, natural polymers typically show poor mechanical properties compared with synthetic polymers, as well as low processing ability and poor reproducibility due to their inconsistency in purity and molecular weight shown from different samples [92].

The collagen-based biomaterials are the most obvious and common choice for musculoskeletal tissue engineering. As a major component in ECM of the connective tissue, collagens represent 25% total protein in the body, with low immunogenicity, easy modification, and high biocompatibility characteristics [93,94]. However, as collagens are purified from animal tissues, the extracted collagens require removal of foreign antigens to avoid disease transmission. A purified collagen sample usually requires crosslinking to improve its mechanical strength, which can also slow down the degradation rate. However, the mechanical strength of a crosslinked collagenous scaffold still fails to match those of native tendon/ligament tissues with either physical or chemical crosslinking methods, and the scaffold is still associated with a relatively fast in vivo degradation rate [10,73]. Bellincampi et al. fabricated collagen fibers and cross-linked them to produce collagen fibrous scaffolds for seeding rabbit ACL or skin fibroblasts for autogenous reconstruction of the ACL in the knee [95]. They crosslinked ovine dermal collagen fibers extruded through a polyethylene tubing and formed in a fiber-formation buffer by ultraviolet light to increase the tensile strength of the fibers. This was followed by aligning two hundred crosslinked fibers in parallel to form a scaffold with 3-mm diameter. The cells seeded in the scaffold can survive for at least 6 weeks post-implantation with high cell viability, and the scaffold can be completely reabsorbed within 6 weeks [95]. Similarly, rabbit ACL or PT fibroblasts were seeded in a crosslinked collagen fiber scaffold to investigate cell adhesion and viability [96]. The PT fibroblasts can proliferate faster than the ACL fibroblasts, but both fibroblasts showed a 10-fold higher collagen synthesis rate in the scaffold than on a tissue culture plate, and the cells remained viable after autogenous transplantation into a knee joint [96]. For guided tissue regeneration of ruptured ACL, the cells isolated from an ACL must retain the ability to migrate into an implanted scaffold for repair of ligament rupture. For this purpose, Murray et al. confirmed that ACL fibroblasts isolated from ruptured human ACLs can retain their ability to migrate into collagen-glycosaminoglycan scaffolds in vitro, with cellular growth and expression of a contractile actin isoform [97]. Numerous cross-linking methods for collagens have been investigated to enhance the mechanical properties of collagen-based scaffolds [98]. Although the mechanical properties of crosslinked collagen scaffolds can be improved, they are still incapable of mimicking the strength of a natural ACL [99,100]. Toward this, Uquillas et al. optimized the cross-linking parameters to improve the mechanical properties of collagen scaffolds fabricated from electronically aligned collagen threads [101]. The mechanical properties of the collagen thread scaffold after crosslinking with 2% genipin were significantly improved to mimic the mechanical strength of a native tendon tissue. The cell adhesion and proliferation rate of human mesenchymal stem cells seeded in the scaffold also increased after scaffold cross-linking. Similarly, Younesi et al. formed a 3D textile scaffold from collagen-based threads to mimic the mechanical properties and load-displacement behavior of native tendons [102]. Topographically, the scaffolds made from collagen threads showed a densely packed texture and aligned substrate structure. The scaffolds can stimulate tenogenesis, with seeded mesenchymal stem cells showing up-regulated expression of tenogenic markers such as tenomodulin, COL I, and cartilage oligomeric matrix proteins.

The silk fibers from the cocoons of the mulberry silkworm *Bombyx mori* were used in most of the research works using silk as a raw material for scaffold fabrication, which is particularly promising in the musculoskeletal field [103,104]. The exceptional high strength and toughness provided by silk, when compared to other natural and synthetic polymers, make it an interesting choice for regeneration of tendons and ligaments [105,106,107]. The silk fibers will lose their tensile strength with time in vivo and complete degradation by proteolysis is expected within two years. This degradation rate matches the mechanical strength requirement of a scaffold for tendon and ligament regeneration. The strength requirement met by a silk scaffold can also be gradually transferred to a neo-tissue during scaffold degradation [108]. Furthermore, silk scaffolds can enable the attachment and proliferation of a variety of primary cells and cell lines, including bone marrow stem cells (BMSCs) and fibroblasts [108]. Liu et al. fabricated a hybrid scaffold by impregnating microporous silk sponges to a knitted silk fiber scaffold [109]. They studied the cellular responses of rabbit ACL fibroblasts (ACLFs) and BMSCs in the scaffold for ACL tissue engineering. The BMSCs proliferated faster than the ACLFs, and the expression of ligament phenotypic marker genes was significantly upregulated for BMSCs in comparison with ACLFs. The BMSCs also produced more ligament-related ECM than ACLFs, leading to the conclusion that BMSCs are a preferred cell source for the regeneration of ACL using silk-based scaffolds. Similarly, a scaffold made from bundles of silk fibers was found to be suitable for the tissue engineering of ACL, which can also match the mechanical requirements of a native human ACL [110]. Besides unique mechanical properties, the biocompatibility and slow degradability of this silk-based scaffold also provide a suitable milieu for differentiation of BMSCs toward the ligament lineage. Tissue engineering scaffolds based on silk can be fabricated using a variety of textile techniques, such as twisting, braiding, and knitting. The braiding of silk fibers into a wire rope-like structure can form a scaffold. This was reported by Teuschl et al., who used this method to prepare a silk ACL graft for seeding autologous stem cells [111]. The cell/scaffold construct was effective in stimulating ACL regeneration in a sheep model when tested in vivo, and the elastic modulus of the implanted construct was equivalent to that of a native ovine ACL [111]. Although silk fibroin is a good substrate for adhesion, proliferation, and differentiation into a ligament lineage of BMSCs, Chen et al. further modified a silk film with short polypeptides, and the modified silk film permitted higher rates of ECM production, adhesion, and proliferation of BMSCs compared with pristine silk matrices [112]. A few studies investigating silk-based ACL grafts in large animal models have generated encouraging results [107,113]. Fan et al. prepared a scaffold by rolling a knitted microporous silk mesh around a braided silk cord [107]. The mesenchymal stem cells seeded in these scaffolds proliferated and differentiated into fibroblast-like cells with expression of COL I, COL III, and tenascin C genes. The cell/scaffold construct can be implanted into a larger animal pig model to regenerate the ACL. The regenerated ACL neo-tissue can compensate for the tensile strength loss from degradation of the scaffold 24-week postoperatively, and robust cell proliferation and fibroblastic differentiation of stem cells were found [107]. Liu et al. developed a combined scaffold for ACL tissue engineering by incorporating microporous materials into a knitted silk scaffold [109]. After seeding with BMSCs or ACL fibroblasts, the scaffold was implanted into rabbits and the BMSCs demonstrated better cell proliferation and glycosaminoglycan synthesis, as well as ligament-related ECM marker gene expression and protein synthesis, when compared to ACL fibroblasts 4-week post-implantation [109].

Using the collagen derivative gelatin, Yang et al. [114] fabricated injectable gelatin microcryogels (GMs), which showed a microporous structure and good pore connectivity as well as excellent biocompatibility toward ADSCs. The microporous gelatin microcryogel scaffold could promote cell attachment and proliferation. They repaired Achilles tendon defects with ADSCs + GMs, ADSCs, GMs, or a blank control and found both the ADSCs + GMs and ADSCs groups enhanced the macroscopic appearance and the biomechanical properties of repaired tissue without inducing an unfavorable immune response. Other than suggesting that ADSCs have stimulatory effects on Achilles tendon healing, the injectable microgel scaffold can provide a less traumatic injectable cell/scaffold delivery method for the repair of ruptured Achilles tendons [114].

Hyaluronic acid (HA) or hyaluronan is a non-sulfated, linear GAG made up of repeating disaccharide units of D-glucuronic acid and N-acetyl-D-glucosamine. With a backbone containing 5000–30,000 sugar molecules, this biopolymer occurs naturally and provides a good starting materials for fabricating scaffolds for ACL replacement [115,116,117]. As the natural form of HA is in a gel from with rapid degradation rates, chemical modification of HA to enhance its processability and adjust its biodegradation rate have been suggested [117]. Using esterified sodium hyaluronate, Cristino et al. fabricated a HA-based scaffold with a multilayered knitted cylindrical array of fibers, which were disposed transversally to the longitudinal axis externally and disposed parallel to the central axis of the scaffold in the center [117]. After short-term cell culture with mesenchymal stem cells, the seeded cells completely wrapped the fibers in the scaffold and expressed CD44, which is an important receptor for interaction with HA in the scaffold. The cells also secreted proteins responsible for the functional characteristics of ligaments, including COL I, COL III, laminin, fibronectin, and actin, but did not secrete collagen type II and bone sialoprotein [117]. Alternatively, coating scaffolds with HA is another venue that can be explored for the tissue engineering of tendons and ligaments, where the controlled release of growth factors from the HA gel could also be accomplished. Funakoshi et al. coated chitosan fibers prepared by wet spinning with HA gel [118]. The HA coating could significantly increase the mechanical properties of the fibrous scaffolds as well as enhance the in vitro biological response of cultured rabbit fibroblasts in terms of cell attachment, proliferation, and production of COL I. With HA hybridization with the chitosan fiber sheet stacked scaffold, ECM production, cell adhesion, and proliferation rates increased when compared to controls using chitosan fiber scaffold by seeding with rabbit fibroblasts. On day 1, more cells were found in hybrid HA/chitosan scaffolds from DNA content analysis over chitosan scaffolds, and immunostaining signal and mRNA level of COL I, as well as mechanical properties, were clearly predominant in the hybrid scaffolds using HA coating [118]. Recently, Chen et al. developed a HA/PCL core–shell fibrous membrane to act as an anti-adhesive barrier by preventing fibroblast attachment, penetration, and focal adhesion while providing lubrication for smooth tendon gliding during post-surgical tendon repair [119]. This membrane scaffold could promote tenocyte migration and fasten tendon healing, indicating that HA hybridization with biodegradable synthetic polymers can improve the biological properties of cultured tenocytes.

#### 2.1.3. Natural Polymer Blend

It is challenging to find a single natural or synthetic polymer that can meet all the requirements as a scaffold material [120]. A composite material can combine the beneficial properties from its constituents, with the possibility to show synergistic features not found in pristine single materials. Therefore, a composite scaffold that can mimic the complex structures of tendons or ligaments is expected to show improved biological, biophysical, and mechanical properties for tendon and ligament tissue engineering [14,90,121,122]. Polymer composite materials, which are also known as polymer-based nanocomposite materials, have emerged as promising options for load-bearing applications in various fields [123,124]. The use of composite scaffolds has been reported to completely restore the functions of a tendon or ligament tissue to a state before injury, using simple surgical procedures with minimal patient morbidity [14,90,98,125]. Although scaffolds made from collagen are suitable for cell attachment and cell growth, their inadequate mechanical properties prevent their practical use in tendon and ligament regeneration. Similar challenges in mechanical properties have been found for various biological materials, such as gelatin, HA, and silk, although these biological materials can support cell attachment and cell growth [106,126,127]. Specifically, collagen-based scaffolds are usually in a gel or sponge form with low mechanical strength, which restricts their use in the regeneration of load-bearing tendons. To solve the inherent deficiency in mechanical properties of pristine collagen scaffolds, Panas et al. fabricated different collagen/silk composite fiber scaffolds and demonstrated that a scaffold contains 14% (*v*/*v*) silk and 86% (*v*/*v*) collagen can provide similar or even higher ultimate tensile stress in comparison with human ACL [128]. Walters et al. used a braid-twist design to fabricate a COL I fiber-based scaffold, which after crosslinking and adding gelatin was used for a mechanical property evaluation and cellular response study using rat ligament fibroblast cells [129]. The crosslinked scaffolds with gelatin displayed a Young’s modulus and an ultimate tensile strength closest to those of human ACL, in addition to increased cellular activity [129]. Chen et al. fabricated knitted silk scaffolds impregnated with freeze-dried collagen sponges for the culture of BMSCs [130]. In a rabbit medial collateral ligament repair model, the defect treated with a silk/collagen scaffold improved structural and functional ligament repair, forming collagen fibrils and collagen fibril assembly with a larger diameter than those treated with a silk scaffold. In a follow up study, the knitted silk-collagen sponge scaffold was used for the culture of human embryonic stem cell-derived mesenchymal stem cells (hESC-MSCs) under mechanical stimulation in vitro [131]. The hESC-MSCs exhibited tenocyte-like morphology and upregulated expression of tendon-specific gene markers and other mechano-sensory molecules. After ectopic implantation in rats, the cell/scaffold construct displayed more aligned cells and larger collagen fibers, as well as higher tendon regeneration ability from histology and mechanical property characterization.

The physiologic benefits of HA, such as improved cellular adhesion and proliferation and anti-inflammatory properties, may improve ligament tissue regeneration. Using this benefit from HA, Majima et al. fabricated composite chitosan/HA fibrous scaffolds containing different weight percent of HA. They showed that the scaffold made from 0.1% HA provided adequate biodegradability and biocompatibility [132]. In vivo animal experiments using fibroblasts from rabbit Achilles tendon in the chitosan/HA scaffold revealed less inflammation induction in vivo, and the mechanical properties of regenerated tendon/ligament tissues could stabilize the joint. A silk/collagen scaffold with collagen microsponges formed within a knitted silk scaffold has been shown to be effective for tendon and ligament repair [133,134,135,136]. Bi et al. used a silk/collagen scaffold for ACL reconstruction and found good infiltration of fibroblast-like cells in the graft. The stiffness of the graft was much higher than that of an autograft 16-week post-operation [133]. Zheng et al. developed an aligned collagen/silk scaffold and evaluated its biomechanical performance after implantation in a rabbit massive rotator cuff tear model [136]. The scaffold had similar 3D alignment of collagen fibers found in natural tendons and provided superior mechanical properties. The seeded tendon stem/progenitor cells displayed well-aligned spindle morphology in the scaffold, with intercellular contacts and ECM deposition after 7-day in vitro culture. The rotator cuff tendon defects in rabbits were repaired with this aligned collagen/silk scaffold, which could regenerate a neo-tendon tissue with a failure force 13-fold higher than that provided by a collagen sponge 12-week post-implantation. The regenerated tendon tissue also displayed abundant collagen fibrils, resembling the native microstructure but with larger diameters and better alignments [136].

To evaluate the angiogenesis ability of a composite silk/collagen/HA scaffold, Seo et al. prepared a knitted silk scaffold and fabricate it into a composite scaffold by freeze drying a collagen-HA solution in the scaffold for ACL regeneration [137]. By seeding human ACL cells in the scaffolds, the composite scaffold exhibited the highest cell attachment and proliferation rates, but no difference in the immune response was found between the two scaffolds by T-lymphocyte culture in vitro. By implanting the scaffold as a graft for ACL repair in the knees of dogs, inflammatory tissue response at implant site was noted in both scaffolds from gross examination. The composite scaffold-grafted group revealed granulation tissues consisting of fibroblasts and collagen fibers from histological study, in addition to new blood vessel formation from CD31 staining. On the other hand, no blood vessels, cells, or collagens were found in the pristine silk scaffold, suggesting that the lyophilized collagen-HA in silk fibers can enhance cell migration and new blood vessel formation in vivo [137]. The fibrin hydrogel scaffold formed from fibrin protein is biodegradable, biocompatible, and has a porous fibrillar structure to treat tendon injuries [138]. However, both biomechanical properties and in vivo stability of a fibrin hydrogel scaffold remain a challenging task for its application in tendon tissue engineering. To solve this problem, nanostructured fibrin/collagen and fibrin/agarose hydrogels were demonstrated to provide enhanced healing efficacy in the surgical repair of rat Achilles tendon ruptures with better functional, histological, and histochemical results [139,140]. A summary of composite scaffolds from natural polymer blends for tendon and ligament tissue engineering is included in Table 1.

#### 2.1.4. Synthetic and Natural Polymer Blends

Other than blending natural polymers, a key strategy for producing hybrid scaffolds in ligament and tendon regeneration has been the combination of natural and synthetic polymers. Natural and synthetic polymers each have their own advantages and disadvantages. Although natural polymers are biocompatible and can promote cell proliferation/adhesion and mimic the natural ECM, they show fast degradation and low mechanical strength [55]. On the other hand, synthetic polymers show controllable physico–chemical characteristics and good mechanical strength, but low biocompatibility. Hence, a synergetic effect between natural and synthetic polymers may occur by combining them as scaffold materials, with the expectation that a balance between biological properties and mechanical performance could be accomplished for tendon and ligament regeneration [59,141,142]. A hybrid nanofibrous membrane scaffold with a PCL shell and a HA + Ag nanoparticle (NP) core was produced to prevent peritendinous adhesion by Chen et al., anticipating that HA can provide effective lubrication during tendon regeneration while Ag NPs can provide antibacterial activity [143]. Rabbit fibroblasts grown on a PCL/HA + Ag NPs nanofibrous membrane showed very high viability to discount the possible cytotoxicity from Ag NPs. In vivo studies with a rabbit flexor tendon repair model indicated that the PCL/HA + Ag NPs membrane can reduce peritendinous adhesion along with an increased mechanical strength of healed tendons [143]. The same group used random and aligned electrospun PCL/silk fibroin nanofibrous scaffolds for the culture of rabbit dermal fibroblasts in vitro, followed by in vivo study with a cell/scaffold construct to repair Achilles tendon defects in rabbits [63]. Silk fibroin can promote cell proliferation and up-regulate the gene expression of tendon marker proteins to a higher extent than that provided by fiber alignment alone. The aligned PCL/silk construct can generate neo-tendon tissues with 60.2% tensile stiffness and 81.3% ultimate load of those found in native tendons with increased deposition of COL I and tenascin C.

In another study, Saatcioglu et al. fabricated electrospun PCL/chitosan fibrous scaffolds by blending 1, 3, or 5% (*w*/*w*) chitosan with 10% (*w*/*w*) PCL for ligament regeneration and evaluated their mechanical properties and cellular response [144]. The addition of chitosan into PCL fibers can increase the fiber diameter in the scaffold in addition to its swelling and degradation behaviors. The attached mesenchymal stem cells showed a network-like structure in the scaffolds, and adding 3% (*w*/*w*) chitosan to PCL fibers provided the best scaffold for cellular response as well as the highest tensile strength from tensile testing [144]. Similarly, Leung et al. investigated aligned chitosan/PCL nanofibers for tendon regeneration using differentiated human BMSCs after conjugating transforming growth factor-β3 (TGF-β3) [145]. The BMSCs in the aligned nanofiber scaffold proliferated and showed an elongated morphology along the fiber orientation, showing upregulated expression of tenogenic maker genes and collagen production compared with tissue culture plates, chitosan/PCL films, and random chitosan/PCL nanofibers. They concluded that physical cues from the alignment of chitosan/PCL nanofibers and biological cues from TGF-β3 can work synergistically for effective differentiation of BMSCs into tenogenic progenitor cells to manage tendon defects [145]. Domingues et al. reported the use of cellulose nanocrystals (CNCs) as reinforcing agents in aligned and random electrospun PCL/chitosan fibrous scaffolds [141]. The incorporation of small amounts of CNCs into the nanofibrous bundles could remarkably elevate their biomechanical parameters to the range shown by native tendon or ligament tissues. Other than providing mechanical support, the aligned PCL/chitosan/CNCs nanofibrous scaffold could provide tendon structure-mimicking topographic cues, a key feature for maintaining tendon cell phenotype [141]. A braided multiscale fibrous scaffold consisting of aligned PCL/collagen/bFGF nanofibers was also fabricated by Jayashree et al. to mimic a native tendon structure [56]. Rabbit primary tenocytes cultured in the multiscale braided scaffold showed higher cell proliferation rates and enhanced expression of tenogenic markers in contrast to controls without bFGF. When subjected to dynamic stimulation in vitro, enhanced cellular proliferation and tenogenic marker expression were found when compared with the static control.

Xu et al. cultured tendon-derived stem cells (TDSCs) in an electrospun poly(L-lactide-co-ε-caprolactone)/collagen nanoyarn scaffold under mechanical stimulation for tendon tissue engineering [146]. They reported well proliferated TDSCs associated with good tendon ECM gene expression and protein synthesis rates when cells were cultured in the scaffold in vitro under mechanical stimulation. Interestingly, after implantation into nude mice for mechanical stimulation in vivo, the TDSCs showed long-term survival and neo-tendon formation. Furthermore, the TDSCs/scaffold construct after dynamic culture in vitro was used successfully to repair injured tendons in a rabbit patellar tendon defect model, where neo-tendon tissues with enhanced production of tendon-related proteins and good mechanical properties were produced in vivo [146]. Darshan et al. recently fabricated suture-embedded spiral wound aligned gelatin/PCL/heparin nanofiber scaffolds [38]. After immobilizing bFGF by bio-affinity to heparin, the scaffold was used for the culture of rabbit tenocytes and the cell/scaffold construct after in vitro culture was used to repair rabbit Achilles tendon defects. The seeded tenocytes showed good cell proliferation, upregulated tenogenic gene expression, and enhanced tendon ECM protein production in vitro, and successful tendon repair was demonstrated in vivo with the cell/scaffold construct.

To replicate a hierarchical multi-tissue transition with change from a mineralized to a non-mineralized tissue at the tendon-to-bone interface, Calejo et al. fabricated hybrid scaffolds for tendon repair [147]. They prepared a wet-spun fibrous scaffold consisting of two parts: PCL/gelatin aligned microfibers to mimic the tendon tissue and PCL/gelatin/hydroxyapatite random microfibers to mimic the bone tissue. The human ADSCs seeded to the PCL/gelatin aligned microfibers showed highly aligned morphology resembling native tenogenic organization and produced tendon ECM molecules. In contrast, cells in the PCL/gelatin/hydroxyapatite part presented a more random cytoskeleton orientation and only produced a osteogenic-like matrix. By assembling the PCL/gelatin and PCL/gelatin/hydroxyapatite microfibers, this fibrous scaffold formed with a continuous topographical and compositional gradient is expected to mimic the structural characteristics at the tendon-to-bone interface [147].

A modified PLGA/silk hybrid scaffold using knitted silk scaffolds and electrospun PLGA nanofibers was developed by Sahoo et al., who assessed its feasibility in ligament/tendon tissue engineering in vitro [148]. The mechanically robust nano-micro scaffolds were assembled from degummed knitted silk scaffolds coated with an intervening adhesive layer of silk solution, followed by direct electrospinning PLGA nanofibers onto the silk scaffold. Taking advantage of the slow degradation rate of silk, which can compensate for the early degradation behavior of PLGA, the hybrid scaffold showed improved mechanical properties and provided continued support to an injured ligament/tendon tissue during tissue regeneration, with the seeded BMSCs cells showing high cell viability and proliferation rates. A follow-up study from this group created a similar PLGA/silk hybrid scaffold but coated the knitted silk microfibers with bFGF-releasing electrospun PLGA fibers [149]. The feasibility to use it for ligament/tendon repair was evaluated in vitro using rabbit bone marrow stem cells, which demonstrated good cell viability in the scaffold. Most importantly, the released bFGF from nanofibers in the hybrid scaffold could promote cell growth and induce gene expression of ligament/tendon-associated ECM proteins, as well as increasing collagen production and scaffold mechanical properties [149].

Full et al. prepared PLGA/COL I/polyurethane (PU) scaffolds by electrospinning for ligament tissue engineering with two PLGA polymers (50:50 and 85:15) and determined its effects on scaffold mechanical properties and cell adhesion [150]. The 50:50 PLGA scaffold showed similar tensile properties to those of knee ligaments, in contrast to weaker tensile properties shown by 85:15 PLGA scaffolds. The aligned fiber scaffold also showed improved tensile properties compared with the random fiber scaffold. By introducing COL I into the fibers, human fibroblasts could attach firmly to the fiber surface and proliferate within the scaffold, as is usually necessary for fibrous scaffolds prepared only with synthetic polymers. Manning et al. fabricated a hybrid scaffold by loading ADSCs and platelet-derived growth factor BB (PDGF-BB) in heparin/fibrin hydrogel and layered it with an electrospun PLGA nanofibrous membrane [151]. The natural polymer hydrogel part of the scaffold allowed for delivery of the growth factor and cells, while the electrospun PLGA backbone provided structural integrity for implantation. From in vitro study, good cell viability and sustained PDGF-BB release was observed. In vivo studies with a flexor tendon defect model created in large animals found only mild immune response from histology during tendon repair using ADSCs. A summary of composite scaffolds from synthetic and natural polymer blend for tendon and ligament tissue engineering is included in Table 2.

### 2.2. Fabrication of Fibrous Scaffolds by Electrospinning

**Figure 5:**
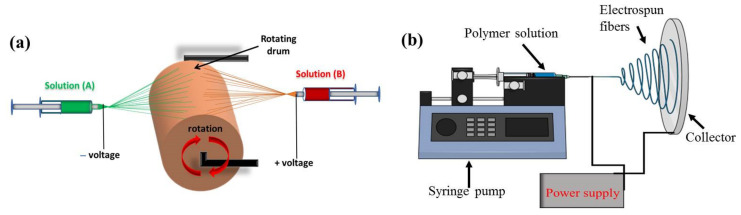
A schematic diagram of the electrospinning process where a rotating drum (**a**) or a static collector (**b**) was used for collecting aligned or random fibers.

The electrospinning technique has been widely exploited for the fabrication of tissue engineering scaffolds [69]. It can produce random or aligned fibers from natural and/or synthetic polymers, with fiber diameters ranging from submicron to micrometer scale (Figure 5) [153]. Usually, electrospun nanofibers are deposited randomly on a static collector, but they can also be collected in a consistent manner using a rapidly rotating collector to produce a membrane scaffold composed of aligned nanofibers [154]. A scaffold consisting of aligned nanofibers can provide seeded cells with topographical signals to regulate directional cellular growth as well as inducing higher cell proliferation and differentiation rates during the formation of functional ligamentous and tendinous tissue [155]. Furthermore, the electrospun fibrous scaffold can be prepared to have high porosity but small pore size, allowing for nutrient diffusion but preventing penetration of fibroblasts responsible for post-operative adhesion formation [156]. Notably, electrospinning can prepare different nano/microscale fibrous scaffolds with a complex 3D structure, resembling the natural ECM in tissues, and these have shown great promise for making artificial functional tissues [157,158]. However, electrospun fibrous scaffolds produced traditionally are made up entirely of closed-packed fibers, which can only create a superficial porous structure. A two-dimensional porous surface rather than a three-dimensional porous structure is formed in this case. The small pore size on the surface also inhibits the migration of cells into the interior of the scaffolds and restricts tissue ingrowth [159]. Numerous scientists have examined different nanostructured fibrous scaffolds and their roles in tissue engineering applications, including skin, neural tissue, tendons, ligaments, bone, and cartilage [25,50,51,63,160]. Despite the fact that these newly introduced fibrous scaffolds have been shown to be effective in simulating neo-tissue formation, more study is required to completely understand the cellular responses to complex structures in a scaffold with different pore size distribution and spatial arrangements. As a result, it is important to look at the complex structure of fibrous scaffolds fabricated by electrospinning using different methods.

#### 2.2.1. Multiscale Electrospun Fibrous Scaffold

**Figure 6:**
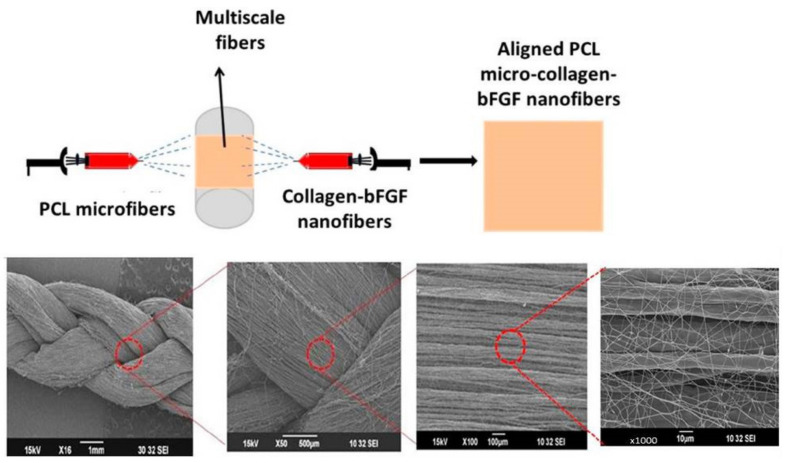
The fabrication and the fibrous structure of a multiscale electrospun fibrous scaffold. Reproduced with permission from [56]. Copyright 2019. American Chemical Society.

A dual electrospinning method combining two extrusion syringes can produce multiscale hybrid scaffolds for tendon and ligament tissue engineering. This approach allows for a multiscale fibrous structure to be incorporated into a single scaffold by controlling the distribution of electrospun fibers with different properties. For instance, Jayashree et al. prepared a braided multiscale hybrid scaffold that contained mixed nanoscale and microscale fibers by dual extrusion electrospinning, with one syringe creating nanoscale fibers and the other providing microscale fibers (Figure 6) [56]. They investigated the cellular response and reported enhanced expression of tenogenic markers by seeded tenocytes during both static and dynamic culture. Park et al. fabricated a stacked hybrid fibrous scaffold containing alternating layers of random and aligned fibers, which provided better mechanical support than a single-layered fibrous scaffold [161]. They reported that the presence of mechano-chemical gradients in the scaffold can help to establish normal loading properties at the tissue interface and promote scaffold integration with bones. Employing two separate electrospinning arrangements, Sensini et al. fabricated multiscale hierarchical scaffolds to replicate the hierarchical arrangement present in natural tendons and ligaments. The scaffold consisted of multiple bundles of aligned electrospun nanofibers, which mimicked the tendon fascicles, wrapped in a sheath of nanofibrous membrane, which replicated the tendon sheath [162]. The morphology of the scaffold could mimic the hierarchical arrangement in natural tendons from X-ray tomographic images. The scaffold also provided mechanical properties (tensile stiffness and toughness) matching those required to replace tendons. Human fibroblasts could attach and proliferate in the scaffold by aligning along the long-axis direction of the electrospun nanofibers for in vivo regeneration of tendons and ligaments. Laranjeira et al. reinforced aligned electrospun nanofiber threads prepared from PCL/chitosan with cellulose nanocrystals and incrementally assembled the nanofiber threads into hierarchical scaffolds to simultaneously mimic the nanotopography, nano-to-macro structure and nonlinear biomechanical behavior of tendons/ligaments. Using human tenocytes and ADSCs, they showed the scaffold can induce anisotropic organization typical of tendon tissues as well as expression of tendon-related markers, indicating the scaffold could prevent the phenotypic drift of tenocytes as well as induce the tenogenic differentiation of ADSCs [163]. To improve the mechanical resilience of the nanofibrous scaffolds, Sensini et al. developed an electrospun bundle of nanofibers to improve the mechanical performance of the scaffolds [164]. By recreating the structure and performance of tendons and ligaments, the aligned hierarchical Nylon 6,6 electrospun assemblies showed comparable yield stress (15.6 MPa) and failure stress (235 MPa) of a tendon or ligament tissue. In a similar study, the same group fabricated electrospun Nylon 6,6 bundles with different nanofiber alignment by controlling the collector speed [165]. The electrospun fibrous scaffold could mimic the stress–strain curve of natural tendons and showed similar transition and inflection points in the stress–strain curve, as well as similar elastic modulus in the linear region.

#### 2.2.2. Co-Axial Electrospun Fibrous Scaffold

**Figure 7:**
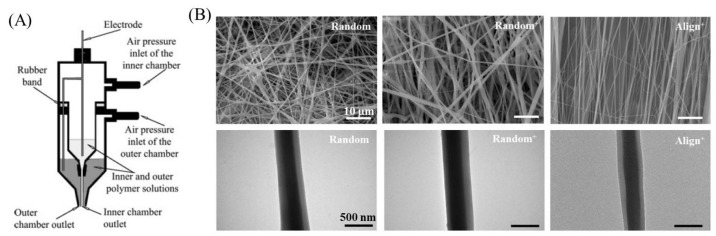
The fabrication (**A**) and fibrous structure (**B**) of co-axial electrospun fibrous scaffolds prepared by electrospinning. Reproduced with permission from [152,166]. Copyright 2003. John Wiley & Sons, Inc.

Co-axial electrospinning technology was used to create core–shell nanofibers and was disclosed by Sun et al. in 2003, where a spinneret made up of two co-axial needles was employed separately to deliver two immiscible spinning solutions during electrospinning [166] (Figure 7A). Full et al. applied co-axial electrospinning to produce random and aligned nanofibers with a PU core and a blend of PLGA/collagen core for ligament tissue regeneration [150]. Mixing different mass ratios of PLGA with collagen in the core solution, they studied the mechanical properties of the fibrous scaffolds, and attachment and proliferation of seeded human foreskin fibroblasts during in vitro cell culture. They found higher mechanical properties of aligned nanofiber scaffolds prepared with 50:50 PLGA/collagen than with 85:15 PLGA/collagen. Moreover, a significant increase in cell attachment in the aligned scaffolds of 50:50 PLGA/collagen was found compared to the other group [150]. Using core–shell nanofibers prepared by co-axial electrospinning, the controlled and sustained release of drugs, growth factors, or nanoparticles could be accomplished by encapsulating or embedding them in the core and/or the shell compartments [167]. Shalumon et al. fabricated core–shell nanofibrous membranes with embedded silver nanoparticles (Ag NPs) in a shell of polyethylene glycol (PEG)/PCL and a core with HA/ibuprofen. The released HA from the core could impart a lubrication effect for smooth tendon gliding and reduce fibroblast attachment. Ibuprofen and Ag NPs can provide anti-inflammation and anti-infection properties, respectively. From in vitro cell culture studies, initial cell attachment and focal adhesion of fibroblasts was effectively reduced on the core–shell nanofiber membrane surface, which also demonstrated minimum cytotoxicity. Simultaneously, the Ag NPs released from the shell could inhibit the growth of both Gram-positive and Gram-negative bacteria. In vivo studies in a rabbit flexor tendon rupture model showed the core–shell nanofibrous membrane can reduce post-operative inflammation and tendon adhesion formation to promote tendon healing [52]. Along this line, Chen et al. fabricated random and aligned nanofibers with a core of HA/platelet-rich plasma (PRP) for growth factor delivery and a shell of PCL for mechanical support (Figure 7B) [152]. The aligned core–sheath nanofibers with PRP (Align^+^) provides the best mechanical properties compared with aligned nanofibers prepared without PRP (Random) and random nanofibers prepared with PRP (Random^+^). The combined effects from growth factors in PRP and fiber alignment in the Align^+^ nanofibrous membrane also led to enhanced proliferation of rabbit tenocytes as well as up-regulated tendon-specific gene expression and increased tenogenic marker protein synthesis. Besides biochemical cues from PRP, the cell-seeded Align^+^ scaffold was mechanically stimulated in vitro under cyclic tensile loading in a bioreactor, by which a shorter tendon maturation time and a higher cell proliferation rate were found with well-preserved tendon phenotype when compared to non-mechanically loaded cell culture conditions. Therefore, topographical cues from nanofibers can be facilely combined with mechanical stimulation to ameliorate the cellular response of tenocytes in a core–sheath nanofiber membrane scaffold for tendon tissue engineering.

#### 2.2.3. Three-Dimensional (3D) Amalgamated Fibrous Scaffold

A variety of methods to fabricate a three-dimensional (3D) amalgamated fibrous scaffold using electrospinning have been attempted to mimic the natural ECM structure of a tendon or ligament, which is made up of a hierarchal structure of aligned collagen fibers at both the micro- and nanoscales [168]. Among these scaffolds, electrospun aligned biodegradable synthetic nanofibers and microfibers can have desirable mechanical properties to regenerate a neo-tissue resembling the native tendon and ligament with alignment, migration, proliferation, and tenogenic differentiation of seeded cells [169]. The 3D electrospun fibrous scaffold could be prepared from aligned nanofiber yarns after electrospinning using advanced textile techniques such as braiding and weaving. Different electrospun PCL scaffolds were fabricated by rolling membranes prepared by melt electrospinning in three bundles, which were subsequently braided and combined with a bone compartment for the development of a bone-ligament-bone construct [170]. The orientation of human mesenchymal stem cells (MSCs) was investigated in vitro and fiber alignment could orientate the cells towards the axial direction of the fibers to highly express tendon-specific genes, as compared with randomly oriented nanofibrous scaffolds where no cell orientation could be found. The final construct with complex geometry could achieve mechanical resilience under cyclic stretching. Considering the inherent low cellularity and vascularity properties of tendon tissue, treatments with aligned nanofibers loaded with thymosin beta-4 was used to improve the biocompatibility, which also significantly upregulated the expression of tendon-specific gene markers, improved cell proliferation, and promoted tenogenic differentiation of human ADSCs [54]. Combining ADSCs with human tenocytes (HTs) and human umbilical vein endothelial cells (HUVECs), simultaneous tri-culture of ADSCs/HTs/HUVECs provided aligned topographic and biomechanical cues to the attached ADSCs.

**Figure 8:**
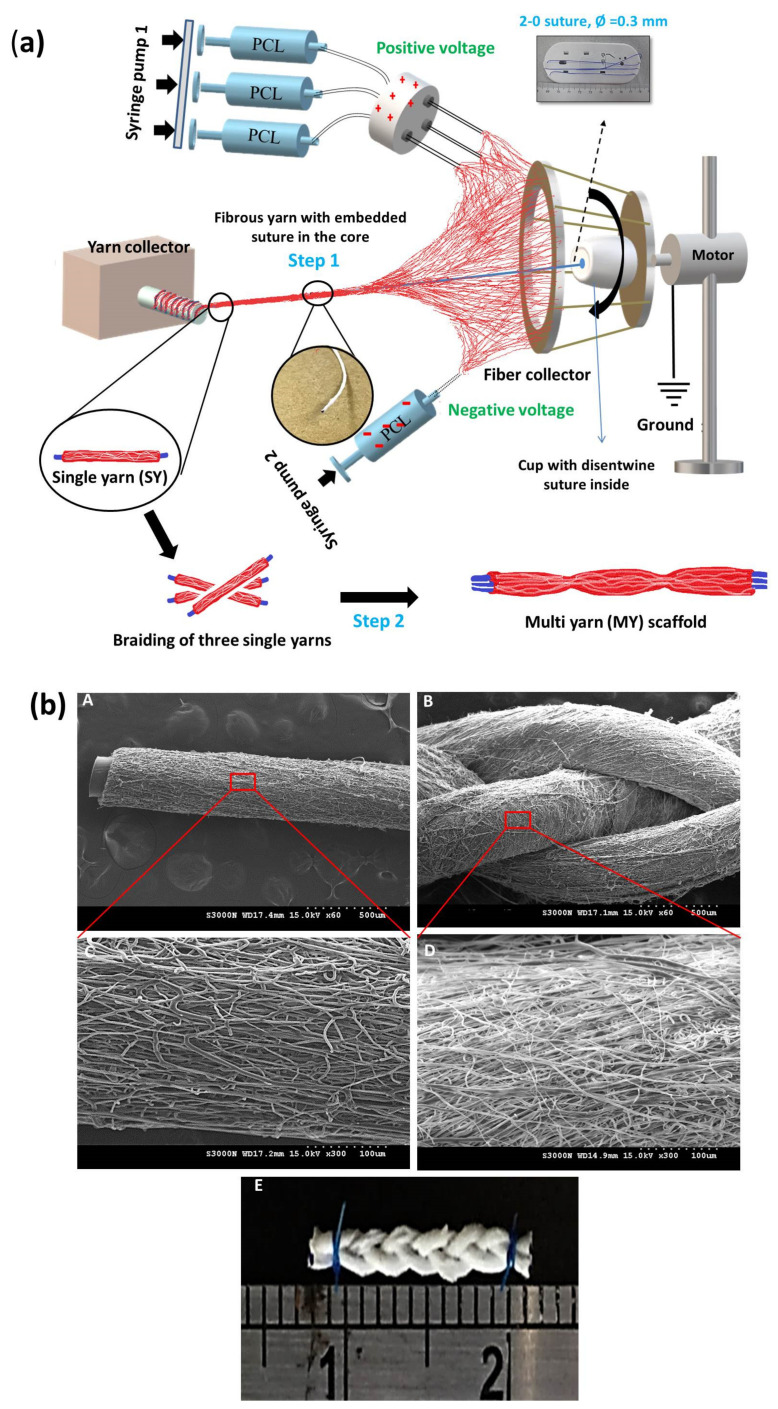
(**a**) The fabrication and fibrous structure of three-dimensional (3D) electrospun fibrous scaffolds. (**b**) The scanning electron microscopy images of suture-reinforced single yarn (SY) covered with fibers (**A**,**C**), and multi yarn (MY) scaffolds from braiding of three SY (**B**,**D**). An optical image of MY scaffold is shown in (**E**). Reproduced with permission from [68]. Copyright 2023. Elsevier.

The bioactive scaffolds utilized in tendon tissue engineering should be able to withstand excessive stresses in addition to encouraging the healing process of injured tendons [58]. Aligned electrospun nanofibers can serve the purpose by providing a structural feature simulating the nonlinear stiffening behavior of crimped collagen fibrils found in tendon tissue and offer mechanical support similar to the highly anisotropic structure found in tendon tissue [168,171]. As scaffolds containing well-aligned ultrafine fibers can exhibit many of the mechanical characteristics of native tendon tissues, a 3D mat of aligned fibers prepared by stable jet electrospinning was used to induce differentiation of human pluripotent stem cells into the tendon lineage [47]. The structure of the scaffold can mimic the microstructure and mechanical properties (Young’s modulus) of native tendons, and seeded stem cells can differentiate into tenocyte-like cells for Achilles tendon regeneration through the activated mechanic-signaling pathway. However, the maximal scaffold tensile strength was only 14.2 MPa, which may restrict its application for tendon regeneration due to the extreme load-bearing sustained by tendon tissues [47]. Following this line, Shalumon et al. prepared a novel 3D fibrous scaffold using the electrospinning technique, in which three suture-reinforced single yarns composed of aligned PCL fibers were braided together to fabricate a multiple-yarn scaffold with good mechanical stability and excellent tensile strength, as well as a fibrous surface topography for cell seeding (Figure 8) [68]. In vitro culture using tendon-derived fibroblasts indicates that fiber alignment can promote cellular proliferation and ECM synthesis, as well as expediting tendon maturation. The cell/scaffold construct after in vitro culture can repair extensor digitorum tendon defects to restore the tendon ECM structure. The use of a 3D electrospun fibrous scaffold as a graft for tendon repair was reported recently [172]. An additively manufactured PCL tubular stent was integrated with two layers of electrospun drug (vancomycin, ceftazidime, and lidocaine)/PLGA and collagen/PCL nanofibrous membranes to treat Achilles tendon rupture. The results from this study indicate the repaired tendon can show increased strength in the stent/drug group compared with the stent group.

## 3. Scaffold Functionalization for Tendon and Ligament Tissue Engineering

### 3.1. Biological Cues Using Growth Factors

Growth factors (GFs) represent the largest group of biomolecules that can induce tenogenesis, and considerable studies have been undertaken to elucidate their roles during tendon healing. GFs can belong to several families, including fibrous growth factor-2 (FGF-2 or bFGF), transforming growth factors beta (TGF-β1, β2, and β3), vascular endothelial growth factor (VEGF), connective tissue growth factors (CTGF), platelet-derived growth factor (PDGF), and insulin-like growth factors-1 (IGF-1) [38,173,174]. GFs not only induce tenogenic differentiation, but also enhance cell growth and expression of gene markers in tendons and ligaments [175]. In response to tendon tissue damage, GFs are released and bind to external receptors on the cell membrane, resulting in intracellular pathways for DNA synthesis and transcriptional expression. This can elicit a direct influence on numerous cellular processes such as cell proliferation, chemotaxis, matrix synthesis, and cell differentiation, all contributing toward the tendon-healing cascade [176,177]. Immediately after tendon injury, activated platelets release GFs from injured tissues, followed by the GF-driven inflammatory cascade to recruit inflammatory cells to the site of damage, followed by more GF production, eventually exacerbating the inflammatory cascade [173]. The tendon cells can align themselves next to the injury area, which helps to activate the cells and produce GFs to promote tendon healing. The optimal milieu for induction tendon healing thus demands the presence of numerous GFs with a precise ratio, and they should be delivered in a well-orchestrated temporal pattern [28,173,174,178]. Although numerous GFs have been identified for this purpose, the precise environment for signal transmission to induce tenogenic differentiation is still largely unknown [56,149,179,180,181].

#### 3.1.1. Basic Fibroblast Growth Factor (bFGF or FGF-2)

Basic fibroblast growth factor (bFGF), which belongs to the family of heparin-binding growth factors, is well recognized for being a strong inducer of angiogenesis and cellular migration. Although in vivo bFGF supplementation can affect early stage rat patellar tendon healing, the prompt inactivation and limited plasma half-life have hindered this effect at the healing site [182]. Considering this, GFs can be integrated into an electrospun fibrous scaffold, which can act as a depot for the sustained release of GFs in addition to replicating the natural ECM structure in ligaments and tendons [180,183,184]. For instance, Sahoo et al. showed bFGF released from a PLGA fibrous scaffold can upregulate the expression of ligament/tendon-specific genes and increase collagen production [149]. Similarly, Jayashree et al. reported that the combination of PCL/collagen/bFGF nanofiber scaffolds with tenocytes can increase the expression of tendon-related markers such as COL I, COL III, and tenascin C [56]. Petrigliano et al. [183] investigated the effects of bFGF in a bFGF-coated porous 3D polymer scaffold and reported results in terms of cell morphology and gene expression [183]. The scaffolds were loaded with different concentrations of bFGF, seeded with cells, and subjected to mechanical stimulation or maintained in a static environment. They reported a dose-dependent stimulatory effect from bFGF, which could be further enhanced in the presence of mechanical tensile strain.

#### 3.1.2. Transforming Growth Factor Beta (TGF-β)

Transforming growth factor beta (TGF-β) has been found to be active in almost all phases of tendon healing by guiding fibroblast migration, neovascularization, and ECM protein production [174]. There are three known isoforms of mammalian TGF-β (β1, β2, and β3), and in many circumstances they do not show distinguishable differences in their effects on cell behavior. To enable binding of TGF-β to its receptors and activation of the intracellular pathways, these isoforms are released as latent precursor molecules and are activated by binding to three different membrane receptors on the surface of cells engaged in the tendon/ligament healing process [185,186,187]. Tellado et al. seeded ADSCs onto TGF-β2 functionalized biphasic silk fibroin scaffolds containing both anisotropic (ligament-like) and isotropic (bone-like) pore structures [175]. They found that pore anisotropy and TGF-β2 functionalization can synergistically increase the expression of tendon/ligament gene markers, with up to a 4-fold increase in the anisotropic (ligament/tendon) region of the scaffold. Such combination strategy of biological and structural cues on stem cell fate may be a promising way for ligament-to-bone regeneration. Chang et al. developed PLLA/PEG electrospun fibrous scaffolds and studied the effects of TGF-β supplementation on long-term matrix deposition. The TGF-β could transform the fibroblast phenotype from proliferative to synthetic and stimulate matrix deposition and enhance collagen production, but showed minimal effects on cell growth [188]. A TGF-β3-immobilized chitosan/PCL nanofiber scaffold was investigated by Leung et al. for tendon tissue engineering, in which rapid and effective differentiation of human BMSCs into tenogenic progenitors was demonstrated [145]. Similarly, Roth et al. studied the bioactivity of scaffold-associated TGF-β3 in vitro by physically adsorbing TGF-β3 to decellularized digital flexor tendon scaffold [189]. Using scaffold-associated TGF-β3 or free TGF-β3 in cell culture medium, similar cell conformation change and tendon-specific gene expression was found, and upregulation of tenascin C and downregulation of other matrix molecules such collagen IA1 and IIIA1 was found, indicating that the bioactivity of TGF-β3 is preserved after binding to the scaffold [189]. The TGF-β1 has been shown to promote cellular migration and proliferation as well as enhancing the production of COL I and COL III in flexural tendon-derived cells [190]. Although all three isoforms of TGF-β affect the formation of ECM proteins in mesenchymal cells and tenocytes, the TGF-β1 stimulates the production of collagen IA1 and IIIA1 the most [191]. However, other studies have found that this effect mediated by TGF-β1 is restricted to tendons only, and other tissues have a different temporal and spatial gene response when stimulated with TGF-β1 [187]. However, Maeda et al. showed that an acute tendon transection injury can result in destabilized organization of tendon ECM structure, with the excessive release of TGF-β and ultimately the death of tenocytes [192]. This mortality of tenocytes can be prevented by inhibiting the TGF-β1 activity. Although a number of in vitro and in vivo studies have shown that inhibiting TGF-β1 production may prevent adhesion formation and expand the range of motion in flexor tendons, some studies have also demonstrated a decrease in the mechanical strength of healed tendon tissues [193,194].

#### 3.1.3. Platelet-Derived Growth Factor BB (PDGF-BB)

Platelet-derived growth factors (PDGFs) are a class of dimeric polypeptide isoforms that are made up of three structurally identical subunits. Because of their well-known chemotactic, mitogenic, and angiogenic properties, PDGFs have been studied as a GF to help tendon healing after damage or rupture to tendons [195]. The PDGF-BB is the most effective chemotactic factor for mesenchymal stem cells when compared to other dimer isoforms (AB, AA, CC, and DD) of PDGF. This has elicited interest centered on the effective delivery of PDGF-BB for wound healing and tissue regeneration with diverse biomaterial formulations. Furthermore, PDGF can promote the production of other GFs, such as insulin-like growth factors, to aid in tendon repair [174,195]. A PDGF-BB immobilized heparin/fibrin hydrogel can coat PLGA fibrous scaffolds with sustained GF release behavior, which is beneficial for the cellular activity of ADSCs in vitro and the repair of intrasynovial flexor tendon defects in a canine model [151]. The cellularity and vascularity of the injured tendons were increased following the transplantation of a cell/PDGF-BB-incorporated scaffold construct [151]. To improve the biomechanical properties of repaired tendons by delivery of GFs at the injured site, Evrova et al. incorporated PDGF-BB into double-layered coaxially electrospun tubes made from biodegradable polyester/urethane block copolymer [196]. The PDGF-BB-incorporated scaffold was used as a graft to regenerate tendon tissue and to improve tendon healing in an Achilles tendon full laceration model in rabbits. Three weeks after implantation, PDGF-BB delivered from the scaffolds did not lead to the hyperproliferation of pro-fibrotic cells and upregulation of α-smooth muscle actin expression at the wound site. Nonetheless, the tensile strength of the healed tendons increased 2-fold using scaffold-based delivery of PDGF-BB, and increased production of COL I and COL III but decreased production of fibronectin was found.

A summary of the scaffold-based delivery of growth factors for tendon and ligament tissue engineering is provided in Table 3.

### 3.2. Physical Cues Using Cyclic Tensile Mechanical Stimulation

In tendon and ligament tissue engineering, a growing emphasis is placed on dynamic mechanical stimulation during cell culture, by culturing the cell/scaffold construct in a bioreactor under cyclic tensile loading. It is reported that mechanical stimulation is one of the most important physical cues affecting cell proliferation, gene expression, ECM formation, collagen fiber alignment, and tissue remodeling in tendons and ligaments [197]. Moreover, by conveying mechanical tensile strain to cells, the mechanical signals may activate the adhesion receptors on cell surface and trigger downstream intracellular pathways [198]. However, the effects of mechanical stimulation on cells are usually influenced by the magnitude of strain, duration time, and frequency of tensile loading, and all these factors influence the phenotypic gene expression of mechanically loaded cells during in vitro culture [199]. Furthermore, different kinds of mechanical stimulation might play a different role during cell differentiation. For instance, mechanical compression is advantageous for both osteogenic differentiation and chondrogenic differentiation, but mechanical stretching has been frequently used for tendons to induce tenogenic differentiation [199]. As the main mechanical stimulus for the formation and development of tendons is tension-induced mechanical stretching; the biophysical signal provided from mechanical stretching is expected to provide physical cues to improve the functions of engineered tendons. To this end, the in vitro mechanical stimulation of cells seeded in a scaffold before implantation may have significant implications for treating tendon or ligament injuries, and damaged tendons or ligaments will be regenerated better using this biomechanical signal. However, it should be noted that mechanical stretching may also result in unexpected consequences, such as increasing the diameter of the scaffold, causing elongation of the scaffold, or reducing the mechanical properties of the scaffold. Furthermore, cells, especially stem cells, may undergo early differentiation and apoptosis if they are subjected to excessive mechanical tensile loading [200,201]. It should also be noted that mechanical stretching has been shown to be highly related to the strain value, and only a particular range of strain can promote tenogenic differentiation [202].

Wang et al. studied the effect of uniaxial and biaxial mechanical loading on tendon-derived stem cells (TDSCs) by applying a 6% biaxial or uniaxial cyclic strain at 0.25 Hz, 8 h/day, for 6 days to enhance tenogenic differentiation [203]. They reported better differentiation of TDSCs toward the tenogenic lineage by applying uniaxial loading, judging from tendon-specific marker gene expression and protein production, while biaxial loading causes TDSCs to differentiate into the osteogenic, adipogenic, and chondrogenic lineages. Engebretson et al. also demonstrated that mesenchymal stem cells seeded on human umbilical vein (HUV) under mechanical stimulation at 2% strain, 30 min/day, 0.5 Hz can improve the cellularity in a cell/HUV construct by 37% and the ultimate tensile strength by 33% in 14 days [204]. Although 1% to 15% strain has been shown to be beneficial for tendon differentiation, the strain is usually controlled within 4–8% based on the physiological strain experienced by a tendon in vivo. In earlier studies, dynamic stretching at 10% strain could increase the expression of tendon-related genes such as COL I, scleraxis, and tenascin C. However, further increasing the strain to 15% resulted in decreased production of COL I and tenascin C [198,205]. Consistent with the trend, Chen et al. found the genes and proteins related to tendons, such as COL I and tenascin C, were expressed more in aligned fibrous scaffolds seeded with tenocytes and stimulated at 6% strain [152]. Similarly, Jayashree et al. reported that a multiscale PCL–collagen braided scaffold could increase cellular proliferation and tenogenic marker expression of seeded tenocytes with 5% strain, 0.5 Hz, and 3 h/day mechanical stimulation [56]. Recently, Shalumon et al. showed how different strain rates at 3% and 5% can affect cell growth, gene expression, and protein synthesis in PCL multi-yarn scaffolds [68]. Compared to static cultures, dynamic culture with 5% strain mechanical stimulation can improve the rates of cell proliferation and tenogenic differentiation. Xu et al. revealed that mechanical stimulation under different amplitudes and frequencies leads to distinct effects on TDSCs seeded in electrospun nanoyarn scaffolds, including cell proliferation and tenogenic differentiation [206]. These effects were most pronounced at 0.5 Hz and at 4% cyclic tensile strain. In addition, the signaling pathway analysis showed that although cyclic tensile strain impaired the ECM–receptor interaction, this physical cue strongly upregulated the genes that encoded the regulators of transcriptional activities and transcriptional factors and aided in cell proliferation and differentiation. This transcriptome analysis may provide new insights into the signaling networks and the molecular mechanism when TDSCs are under tensile loading in a fibrous scaffold.

A PCL nanofibrous woven scaffold was used for the tri-culture of human ADSCs/human tenocytes (HTs)/human umbilical vein endothelial cells (HUVECs) under cyclic uniaxial elongation along the direction of nanofibers using 4% strain at 0.5 Hz, 2 h/day, for a total of 12 days. The mechanically stimulated cell/scaffold constructs under dynamic stretch can significantly enhance tenogenic differentiation with increased production of tendon-related proteins and upregulated expression of tendon-specific genes [169]. Using uniaxial stretching, Nam et al. cultured BMSCs in a silicon chamber and uniaxially stretched the cells under different strains in vitro. They found that most tendon-related genes and proteins were expressed under 4, 8, or 12% strain rate and at 0.5 or 1 Hz frequency [207]. The highest cell proliferation rate was found at 4% strain/1 Hz, and the highest collagen production and tenogenic gene expression were found at 8% strain/1 Hz, but no significant increase in both cell proliferation and tenogenic differentiation were found by further increasing the strain or frequency. In general, high strain can induce early cell differentiation and apoptosis, but it can also change the mechanical properties of the scaffold, where excessive elongation can lead to enlarged pore size. In contrast, a strain that is too low might not be able to induce the intended stimulatory effects. The ideal strain rate might also be different in different loading systems under different operating conditions, which demands careful study to tailor to each circumstance [205,208]. However, in scaffold-based physical signaling, most of the stretching frequencies used are lower than 1 Hz in tendon tissue engineering.

Indeed, a collective finding from previous studies underlines that a mechanical stretching frequency of 1 Hz may be the optimal value to generate a favorable cellular response, such as an exceptionally high degree of cell proliferation and tenogenic differentiation, and a diminished beneficial effect was found beyond this value. Nevertheless, a lower frequency may be better when considering the quality of regenerated tendons or ligaments. Additionally, cells can be progressively adapted to the stimulus during the rest intervals, resulting in a reduction in the impact of the applied mechanical stimulation. By including a rest interval, the mechanical sensitivity of stretched cells may be restored and result in a more favorable impact. Several studies found that shorter duration and lower frequency were associated with higher cell proliferation rates. The highest cell proliferation rate was found with mechanical stretching at 0.5 or 1 Hz and 2 or 3 h/day, which resulted in upregulated tenogenic marker expression and high-quality neo-tendon tissue formation under mechanically stimulated in vitro [68,146,169,200,206]. In general, it might be challenging to determine which dynamic stretching parameter is associated with the strongest impact on tenogenic differentiation. Therefore, using a bioreactor that offers the possibility to control several tensile stretching parameters simultaneously is preferred for examining the effect of each stretching parameter individually. Nonetheless, a neo-tendon construct demands appropriate biomechanical stimulation with well-tuned parameters in a biomimetic milieu simulating a native tendon/ligament in the scaffold. To this end, different imaging techniques such as high-resolution X-ray tomography, fluorescent microscopy, and scanning electron microscopy may be used to confirm changes of cellular shape and orientation during dynamic culture. Using these techniques, a study by Sensini et al. showed preferential axial distribution of human fibroblasts in electrospun aligned PLLA/collagen fiber bundles by stimulating at 5% strain and 1 Hz frequency for 1 h [209]. Table 4 provides a summary of the scaffold-based delivery of physical cues from cyclic tensile loading in a bioreactor for tendon and ligament tissue engineering.

## 4. Conclusions and Outlook

Regeneration of tendon and ligament tissues using the tissue engineering principle remains a challenging issue in orthopedic research. Current treatment strategies are unable to fully restore the functions of injured tendons and ligaments to their native states, and repair of injured tendons and ligaments continues to be a clinical problem. Using seeded cells in scaffolds supplemented with scaffold-based biological and physical cues, the tendon/ligament tissue engineering approach may provide a solution to this problem, by providing a milieu for cell proliferation as well as for phenotype maintenance of tendon-derived cells or for tenogenic differentiation of stem cells. However, the tissue engineered tendon or ligament should be endowed with improved mechanical properties and functions over naturally healed tendons or ligaments. By combining biomimicry and manufacturing flexibility, electrospinning is undoubtedly one of the most promising methods to fabricate scaffolds for tendon or ligament tissue engineering. Although nano- to macro-scale scaffolds are discussed in this paper without clear distinction, a novel electrospun 3D scaffold mimicking the hierarchical structure of a tendon or ligament must be developed by integrating the beneficial physico–chemical properties offered by natural and synthetic polymers. The growth factors play a crucial role during regeneration of tendons/tendons; therefore, growth factors related to the development of these tissues must be introduced into the scaffold with controlled release characteristics to provide biochemical cues during the regenerative process. Furthermore, a novel scaffold design for scaffold-based gene therapeutics or delivery of exosome may be a promising approach toward this end and is expected to produce positive outcomes in the future. Mechanical stimulation can provide physical cues to cells loaded in a scaffold, which appears to be an important route to generating functional tendon or ligament tissue in a bioreactor under dynamic culture. However, the operation parameters during cyclic tensile loading, such as strain, frequency, and duration time, must be investigated and optimized. With additional refinement and the combination of these components, tissue engineering will undoubtedly be a very convincing option for tendon and ligament repair. However, the type of cells for seeding in a scaffold should be carefully chosen based on the types of tissue to be regenerated, which is expected not only to enhance tissue healing but also to provide acceptable mechanical performance at the tissue level. The development of a feasible cell delivery system that can temporally and spatially reproduce the normal physiology of native tendons or ligaments may require further investigation; however, the inception of new techniques followed by substantial research for optimization will undoubtedly direct them toward clinical applications in due course.

## Figures and Tables

**Table 1:** Summary of composite scaffolds from natural polymer blends for tendon and ligament tissue engineering.

Type of Polymers	Fabrication Methods	Type of Study	Type of Tissue	References
Collagen/gelatin	Chemical crosslinking	In vitro	Ligament	[129]
Silk/collagen	Freeze-drying	In vitro and in vivo	Ligament	[130]
Silk/collagen	Freeze-drying	In vitro and in vivo	Tendon	[131]
Collagen/silk	Freeze-drying	In vivo	Tendon	[136]
Chitosan/HA	Wet-spinning	In vitro	Ligament	[118]
Chitosan/HA	Wet-spinning	In vitro and in vivo	Ligament/Tendon	[132]
Silk/collagen/HA	Freeze-drying	In vitro and in vivo	Ligament	[137]

**Table 2:** Summary of composite scaffolds from natural polymer blend for tendon and ligament tissue engineering.

Type of Polymers	Fabrication Methods	Type of Study	Type of Tissue	Reference
PCL/HA	Co-axial electrospinning	In vitro and in vivo	Tendon	[143]
PCL/silk	Electrospinning	In vitro ad in vivo	Tendon	[63]
PCL/chitosan	Electrospinning	In vitro	Ligament	[144]
Chitosan/PCL	Electrospinning	In vitro	Tendon	[145]
PCL/chitosan/cellulose	Electrospinning	In vitro	Tendon	[141]
PCL/collagen	Mixed electrospinning	In vitro	Tendon	[56]
Gelatin/PCL	Mixed electrospinning	In vitro and in vivo	Tendon	[38]
PLA/PCL/collagen	Electrospinning	In vitro and in vivo	Tendon	[146]
PCL/gelatin	Wet spinning	In vitro	Tendon-to-bone	[147]
PLGA/collagen/PU	Mixed electrospinning	In vitro	Ligament	[150]
HA/PCL	Co-axial electrospinning	In vitro	Tendon	[152]

**Table 3:** Summary of scaffold-based delivery of growth factors for tendon and ligament tissue engineering.

Scaffold	Tissue Type	Growth Factor	Effects	References
Knitted silk scaffold	Ligament/tendon	bFGF	Upregulate gene expression of ligament/tendon	[149]
PCL/collagen fibrous scaffold	Achilles tendon	bFGF	Enhanced cellular proliferation and tenogenic gene expression	[56]
Gelatin/PCL/heparin scaffold	Achilles tendon	bFGF	Upregulated COL I and tenascin C, improved mechanical properties	[38]
Electrospun PLLA/PEO scaffold	Ligament	TGF-β	Improved mechanical properties	[188]
Biphasic silk fibroin scaffold	Ligament-to-bone	TGF-β2	Upregulated tendon/ligament markers, enhanced cell proliferation, and ECM synthesis	[175]
None	Archiles Tendon	TGF-β1	Upregulated gene expression of procollagen type I and type III	[190]
Decellularized superficial digital flexor tendon scaffold	Tendon	TGF-β3	Upregulation of tenascin C and downregulation of decorin gene, enhanced tendon regeneration	[189]
Hydrogel-coated heparin-PLGA fibrous scaffold	Tendon	PDGF-BB	Enhanced cell proliferation, increased tensile strength	[151]
Electrospun coaxially PU tubular scaffold	Tendon	PDGF-BB	Upregulated COL I and COL III, downregulated fibronectin	[196]

**Table 4:** Summary of scaffold-based delivery of physical cues from cyclic tensile loading in a bioreactor for tendon and ligament tissue engineering.

Scaffold	Cell Type	Parameters	Effects	References
PCL–Collagen braided fibrous scaffold	Tenocytes	5% strain, 0.5 Hz, 3 h/day	Up-regulated tenogenic gene expression	[56]
Human umbilical vein (HUV)	MSCs	2% strain, 0.5 Hz, 30 min/day	Increased tensile strength	[204]
Porous PLCL scaffold	Tenocytes	10% strain, 0.25 Hz, 6000 cycle/day	Increased cellularity, mechanical properties, and tenogenic gene expression.	[198]
Collagen sponge scaffold	BMSCs	15% strain, 1 Hz, 3 days cyclic stretching	Increased cellularity, but COL I and tenomodulin expression did not significantly change.	[205]
PCL/HA/PRP core–shell nanofibrous scaffold	Tenocytes	6% strain, 1 Hz, 3 h/day	Increased gene expression of COL I and tenascin c.	[152]
PCL multi-yarn fibrous scaffold	Tendon-derived fibroblasts	3% and 5% strain, 0.5 Hz, 3 h/day	Enhance cell proliferation and ECM synthesis rates, fasten tendon maturation.	[68]
P(LLA-CL)/Collagen scaffold	TDSCs	2, 4, and 8% strain; 0.3, 0.5 and 1 Hz	Up-regulated gene encoding regulator of transcriptional activities	[206]
PCL nanofibrous woven scaffold	ADSCs/HTs/HUVECs	4% strain, 0.5 Hz, 2 h/day	Increased tenomodulin, COL I, tenascin C, scleraxis, and vascular endothelial growth factor (VEGF) gene expression.	[169]
Knitted silk-collagen sponge scaffold	Human ESCs	10% strain, 1 Hz, 2 h/day	Upregulated expression of tendon-related genes scleraxis, COL I and COL III	[210]
P(LLA-CL)/Col scaffold	Rabbit TDSCs	4% strain, 0.5 Hz, 2 h/day	Enhanced tendon-specific gene and protein expression	[146]
3D PCL scaffold	Human BMSCs	10% strain, 0.33 Hz	Increased COL I, COL III, and scleraxis expression.	[211]
Polyurethane disk scaffold	Human fibroblasts	10% strain, 0.25 Hz, 8 h/day	Increased cell proliferation, COL I, transforming growth factor β1 (TGF-β1), and connective tissue growth factor (CTGF) expression.	[212]
Plain knitted silk scaffold	Rabbit TDSCs	2% strain, 1 Hz	Promote tenogenic differentiation from COL III and decorin production	[136]
Collagen sponge scaffold	Rabbit MSCs	2.4% strain, 1 Hz, 8 h/day	Increased COL I gene expression and linear stiffness of construct	[213]

## Data Availability

The data presented in this study are available on request from the corresponding author.
